# A continuous data driven translational model to evaluate effectiveness of population-level health interventions: case study, smoking ban in public places on hospital admissions for acute coronary events

**DOI:** 10.1186/s12967-020-02628-x

**Published:** 2020-12-09

**Authors:** Hossein Bonakdari, Jean-Pierre Pelletier, Johanne Martel-Pelletier

**Affiliations:** 1 Osteoarthritis Research Unit, University of Montreal Hospital Research Centre (CRCHUM), 900 Saint-Denis Street, R11.412, Montreal, QC H2X 0A9 Canada; 2 Department of Soil and Agri-Food Engineering, Laval University, 2425 rue de l’Agriculture, Québec, QC G1V 0A6 Canada

**Keywords:** Transitional model, Data processing, Computer simulation, Hybrid model, Interrupted time series, Lag time, Nonlinear, Public health

## Abstract

**Background:**

An important task in developing accurate public health intervention evaluation methods based on historical interrupted time series (ITS) records is to determine the exact lag time between pre- and post-intervention. We propose a novel continuous transitional data-driven hybrid methodology using a non-linear approach based on a combination of stochastic and artificial intelligence methods that facilitate the evaluation of ITS data without knowledge of lag time. Understanding the influence of implemented intervention on outcome(s) is imperative for decision makers in order to manage health systems accurately and in a timely manner.

**Methods:**

To validate a developed hybrid model, we used, as an example, a published dataset based on a real health problem on the effects of the Italian smoking ban in public spaces on hospital admissions for acute coronary events. We employed a continuous methodology based on data preprocessing to identify linear and nonlinear components in which autoregressive moving average and generalized structure group method of data handling were combined to model stochastic and nonlinear components of ITS. We analyzed the rate of admission for acute coronary events from January 2002 to November 2006 using this new data-driven hybrid methodology that allowed for long-term outcome prediction.

**Results:**

Our results showed the Pearson correlation coefficient of the proposed combined transitional data-driven model exhibited an average of 17.74% enhancement from the single stochastic model and 2.05% from the nonlinear model. In addition, data demonstrated that the developed model improved the mean absolute percentage error and correlation coefficient values for which 2.77% and 0.89 were found compared to 4.02% and 0.76, respectively. Importantly, this model does not use any predefined lag time between pre- and post-intervention.

**Conclusions:**

Most of the previous studies employed the linear regression and considered a lag time to interpret the impact of intervention on public health outcome. The proposed hybrid methodology improved ITS prediction from conventional methods and could be used as a reliable alternative in public health intervention evaluation.

## Introduction

Due to advances in technology and improvements in recording reliable data and sharing methods, the time series (TS) concept has emerged in many theoretical and practical studies over the past few decades [1]. This concept allows researchers to access the outcome of any phenomenon or intervention, at any time, with minimum cost and effort, and to plan possible solutions and control measures based on the forecasted data [2]. Therefore, improving knowledge about studying TS, preprocessing, modeling and, if needed, post-processing is imperative [3].

In the domain of public health interventions, the interrupted time series (ITS) concept has been widely employed to evaluate the impact of a new intervention at a known point in time in routinely observed data [4–10]. ITS is fundamentally a sequence of outcomes over uniformly time-spaced intervals that are affected by an intervention at specific points in time or by change points. The outcome of interest shows a variation from its previous pattern due to the effect of the intervention. The applied intervention splits TS data into pre- and post-intervention periods. Based on this definition, Wagner et al. [11] proposed segmented regression analysis for evaluating intervention impacts on the outcomes of interest in ITS studies. In this approach, the choice of each segment is based on the change point, with the possible additional time lag in some cases, in order for the intervention to have an effect [12–17]. In addition, for pre- and post-intervention period segments of a TS, the level and trend values should be determined either by linear [17] or nonlinear [6] approaches. Therefore, accurate values of the change point and time lag parameters are essential in segmented regression analysis.

**Fig. 1:**
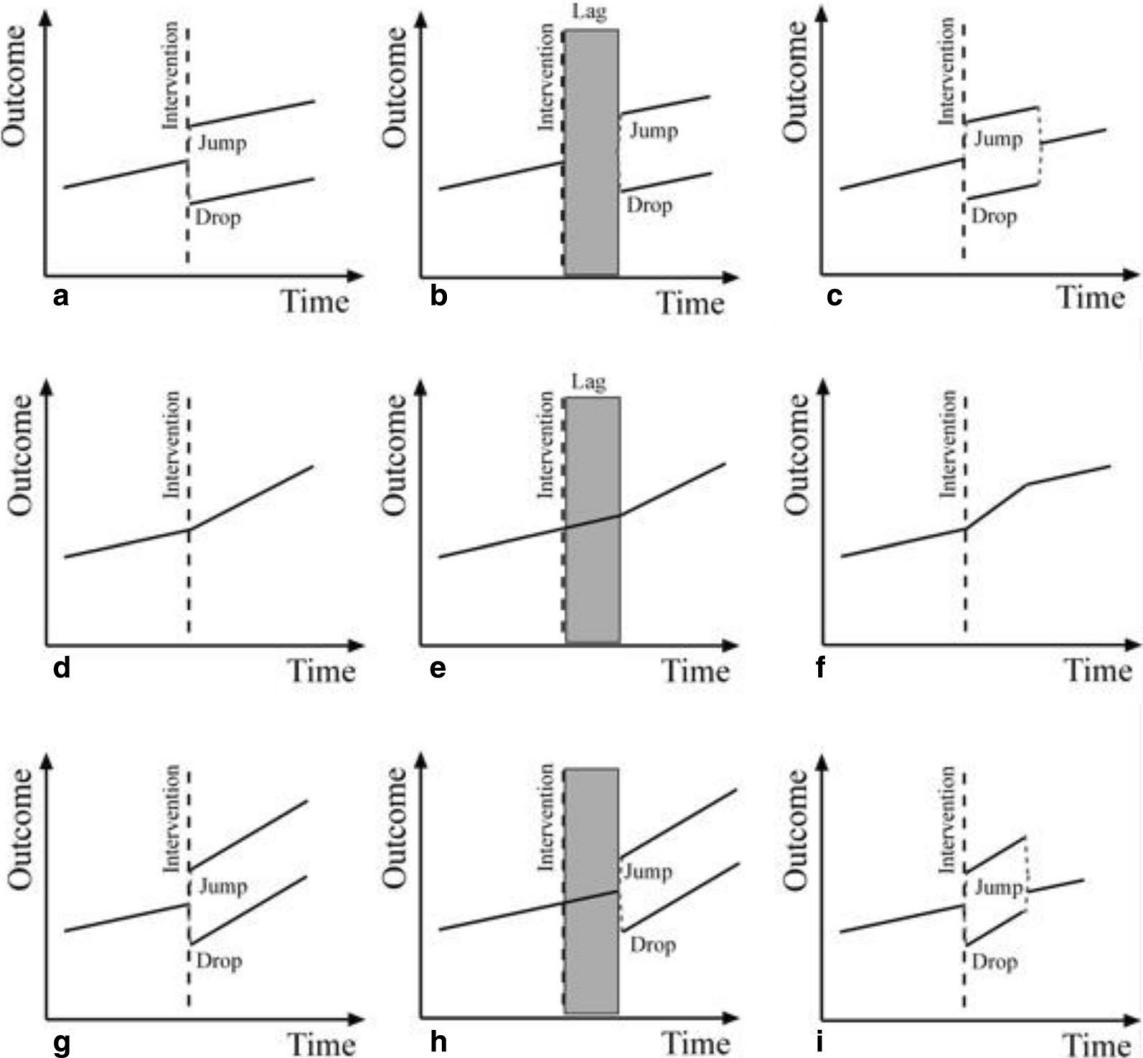
Possible patterns in interrupted time series post-intervention period data analysis

Affecting an intervention at a change point produces different possible outcome patterns in the post-intervention period for both level and trend parameters. Figure 1 illustrates some possible impacts of an intervention on the post-intervention period. As shown in Fig. 1a–c, a change in level (or intercept) may lead to a change in level after a time lag or a temporary level change after the intervention. Other possible patterns are a change in slope (or trend) with a change in slope after a time lag, or a temporary slope change as shown in Fig. 1d–f, respectively. In some cases, a change in both of these parameters could take place as an immediate change, e.g., a change after a time lag or temporary level and slope changes (Fig. 1g–i).

Regardless of the popularity and consensus on using segmented regression-based methods for solving ITS problems, selecting the most appropriate time lag is a challenging task with an important impact on results in this type of modeling. The reason for the delicacy of this task is that there is no specific rule to define the time lag produced between the pre- and post-intervention periods. In some cases, the outcomes of interventions have an unknown delayed response to the implemented strategies and a lag time may occur long after an intervention. However, in ITS modeling, when segmented regression approaches are used, the exact time lag after an intervention should be taken into consideration to guarantee modeling result accuracy and appropriateness. In addition, an undocumented change point seriously complicates ITS analysis. Applying a continuous nonlinear TS method is considered reliable if the ITS analysis can be released from all these fundamental concerns. Therefore, there is a necessity to introduce potential uses of linear, nonlinear or a combination of both models for solving such problems.

Over the past few years, soft computing methods have been employed across domains and have established reliable tools for modeling complex systems and predicting different phenomena in healthcare [18–24]. Among soft computing techniques, the Group Method of Data Handling (GMDH) is a common self-organizing heuristic model, which can be used for simulating complicated nonlinear problems. This evolutionary procedure is performed by dividing a complex problem into some smaller and simpler problems. Based on GMDH, this study proposes a novel methodology of the continuous modeling of an ITS based on data preprocessing. An example of the novel ITS modeling uses a linear-based stochastic model, a nonlinear-based model and an integration of a stochastic and a nonlinear model (hybrid). In order to run the models, certain tests and preprocessing methods are initially applied to the TS to prepare the data for stochastic modeling. It is crucial to investigate the structure of the TS being studied prior to modeling. Therefore, the TS undergoes stationarity testing along with normality testing. After surveying the characteristics of the TS, stationarizing methods appropriate to the TS are used. Then, in case of non-normal distribution, a normal transformation is applied to the stationarized TS. For the second TS modeling approach, the dataset is modeled with an artificial intelligence (AI) method which is, in this case, the Generalized Structure Group Method of Data Handling (GS-GMDH). In the third and final step, a hybrid model that combines the linear and nonlinear results is applied. Finally, the results are compared according to various indices and methods. Therefore, using this method facilitates modeling the ITS continuously, i.e. there is no need to identify the change point and intervention lag time.

### Dataset description

Barone-Adesi et al. [25] carried out an extensive study on the effect of a smoking ban in public places on hospital admissions for acute coronary events (ACEs). In January 2005, Italy introduced legislation that prohibits smoking in indoor public spaces, the goal of which was the reduction of health issues caused by second-hand smoke [25]. Second-hand smoke consists of smoke exhaled by smokers and from lit cigarettes and causes numerous health problems in non-smokers every year, as well as high treatment costs for both patients and the government. The ban was undertaken on 10 January 2005 to confront the growing trend of ACEs and to control this problem.

Bernal et al. [6] used a subset of ACEs data from subjects in Sicily, Italy, between 2002 and 2006 among those aged 0–69 years. They analysed the ITS data by applying segmented linear regression to the standardized rate of ACEs TS associated with the implementation of a ban on smoking in all indoor public places, to calculate the change in the subsequent outcome levels and trends. Based on Barone-Adesi et al.’s [25] assumption, Bernal et al. [6] considered only a level change in ACEs occurring and there was no lag between the pre- and post-segments in the modeling procedure.

Here, we used the dataset from Bernal et al. (Fig. 4, [6]) as an example to illustrate the proposed method’s performance in ITS simulation from real data regarding a health problem; it is not meant to contribute to the substantive evidence on the topic. The dataset employed comprises routine hospital admissions with 600–1100 ACEs. More information about the dataset can be found in the Barone-Adesi et al. article [25].

## Methods

### Preprocessing

Time series are data recorded continuously and based on time to institute a sequence of measures, each of which refers to a time. Thus, the ACE data collected monthly from 2002 to 2006 is a TS. Each TS consists of four terms: jump + trend + period + stochastic component. The first three terms, known as deterministic terms, are calculable and removable. The jump term represents the sudden changes that occur in TS. These changes are detectable as steps in TS plots or by numerical tests. The trend term represents the gradual upward or downward changes that take place during a long period of time; this term is denoted in TS as a linear fitted line. The third deterministic term, the period, represents the periodic alternations in TS, which are seen as sinusoidal variations. Therefore, only the remaining stochastic term is required for use in stochastic or nonlinear modeling. This term is achieved while stationarity (absence of a deterministic term) occurs. Numerous tests and methods exist for investigating and omitting deterministic terms and some are presented below.

In stochastic modeling, two conditions must be met: the first is the stationarity (for details see below, Stationarizing methods); second, the distribution of the TS should be normal. Thus, in order to start stochastic-based modeling, the existence of deterministic terms must be checked, and when present, they should be removed. The Mann–Whitney (*MW*), Fisher, and Mann–Kendall (*MK*) tests are employed to check the jump, period and trend, respectively; the Kwiatkowski–Phillips–Schmidt–Shin (*KPSS*) test to assess the overall stationarity of the TS; and the Jarque–Bera (*JB*) test to check the normality of the TS.

### Trend

A non-parametric test is used to assess the trend term in the studied TS. The *MK* test was developed to detect the gradual changes in TS, both seasonal and non-seasonal. The test equation is as follows [26]:

1 $$ U_{MK} \, = \,\left\{ \begin{gathered} \left( {MK - 1} \right){\text{var}} \left( {MK} \right)^{ - 0.5} \,\,\,\quad \,\,MK > 0 \hfill \\ 0\,\,\,\quad\quad\quad \,\,MK = 0 \hfill \\ \left( {MK + 1} \right){\text{var}} \left( {MK} \right)^{ - 0.5} \,\,\,\,\,\quad MK < 0 \hfill \\ \end{gathered} \right. $$

where *U*_*MK*_ is the standard Mann–Kendall statistic, *MK* is the Mann–Kendall statistic, and *var*(*MK*) is the variance of MK. *MK* and *var*(*MK*) are defined as:

2 $$ MK\, = \,\sum\limits_{i = 1}^{N - 1} {\sum\limits_{j = i + 1}^{N} {{\text{sgn}} \left( {x_{j} - x_{i} } \right)} } $$

3 $$ {\text{var}} \left( {MK} \right)\, = \frac{1}{18}\left[ {N\left( {N\, - \,1} \right)\left( {2N\, + \,5} \right)\, - \,\sum\limits_{g}^{p} {t_{g} \left( {t_{g} \, - \,1} \right)\left( {2t_{g} \, + \,5} \right)} } \right] $$

where *p* is the number of identical groups, *t*_*g*_ is the observation number in the *g*^*th*^ group, *sgn* is the sign function, and *N* is the number of samples.

The (*MK*) test equation for a seasonal trend is expressed as follows:

4 $$ S_{k} \, = \,\sum\limits_{i\, = \,1}^{{N_{k} 1}} {\sum\limits_{j\, = \,i\, + \,1}^{{N_{k} \, - \,1}} {{\text{sgn}} \left( {x_{ki} \, - \,x_{kj} } \right)} } $$

5 $$ SMK\, = \,\sum\limits_{k = 1}^{\omega } {\left( {S_{k} \, - \,{\text{sgn}} \left( {S_{k} } \right)} \right)} $$

6 $$ {\text{var}} \left( {SMK} \right)\, = \,\sum\limits_{k}^{\omega } {\frac{{N_{k} \left( {N_{k} \, - \,1} \right)\left( {2N_{k} \, + \,5} \right)}}{18}} \, + \,2\sum\limits_{i = 1}^{\omega - 1} {\sum\limits_{j = i + 1}^{\omega } {\sigma_{ij} } } $$

7 $$ U_{SMK} \, = \,MK\,{\text{var}} \left( {MK} \right)^{ - 0.5} $$

where *ω* is the number of seasons in a year and *σ*_*ij*_ is the covariance of the statistic test in seasons *i* and *j*.

The trend in the TS is insignificant if $U_{\alpha /2} < \,U_{MK} \, < \,U_{1 - \alpha /2}$ and $U_{\alpha /2} \, < \,U_{SMK} \, < \,U_{1 - \alpha /2}$ and *U*_*α/2*_ and *U*_*1 − α/2*_ are the *α*/2 and 1 − *α*/2 quartiles of the normal cumulative probability distribution. A probability corresponding to the test statistic less than 5% means the absence of a significant trend in the TS.

### Jump

A numerical survey for the jump term in the ACE TS, namely the non-parametric *MW* test, is employed as follows [27]:

8 $$ U_{MW} \, = \,\frac{{\sum\limits_{t = 1}^{{N_{1} }} {\left( {R\left( {g\left( t \right)} \right)\, - \,\frac{{n_{1} \left( {n_{1} + n_{2} + 1} \right)}}{2}} \right)} }}{{\sqrt {\frac{{n_{1} n_{2} \left( {n_{1} + n_{2} + 1} \right)}}{12}} }} $$

where *g(t)* is the ascending ordered ACE series, *R*(*g*(*t*)) is the order of *g*(*t*), and *N*_*1*_$( x_{1}( t )\, = \,\{ {x( 1 ),x( 2 ),  ...,  x( {N_{1}  )} \}} )$ and *N*_*2*_$( x_{2} ( t )\, = \,\{ x( N_{1} + 1), x( {N_{1} + 2} ),.., x( N ) \} )$ are the numbers of sub-series of the main series, such that the sum of these series is equal to main series. If$P_{{\left| {U_{MW} } \right|}}$ is larger than the significant level (in this study α = 0.01), then the jump term is insignificant.

### Period

The significance of periodicity is investigated with the following statistic [28]:

9 $$ F^{*} \, = \,\frac{{N\left( {N - 2} \right)\left( {\alpha_{k}^{2} + \beta_{k}^{2} } \right)}}{{4\left( {\sum\limits_{z = 1}^{k} {\left( {x\left( t \right) - \alpha_{z} \cos \left( {\Omega_{z} t} \right) - \beta_{z} \sin \left( {\Omega_{z} t} \right)} \right)} } \right)}} $$

where F^*^ is the Fisher statistic, *N* is the number of samples, *α*_*z*_ and *β*_*z*_ are Fourier coefficients, and Ω_z_ is the angular frequency. *Α*_*z*_, *β*_*z*_ and Ω_z_ are defined as follows:

10 $$ \alpha_{z} \, = \,\frac{2}{N}\left( {\sum\limits_{t = 1}^{N} {x\left( t \right)\cos \left( {2\pi f_{z} t} \right)} } \right)\,\,\,\;\,z = 1,2,...,k $$

11 $$ \beta_{z} \, = \,\frac{2}{N}\left( {\sum\limits_{t = 1}^{N} {x\left( t \right)\sin \left( {2\pi f_{z} t} \right)} } \right)\,\;\,z = 1,2,...,k $$

12 $$ f_{z} \, = \,\frac{z}{N} $$

13 $$ \Omega_{z} \, = \,\frac{2\pi z}{N}\;z = 1,2,...,k $$

where *f*_*z*_ is the *zth* harmonic of the base frequency.

The periodicity related to Ω_z_ is significant if the critical value of the *F* distribution at a significant level (*F*(2, *N*-2)) is lower than F^*^:

14 $$ F^{*} \, \ge \,F\left( {2,N - 2} \right) $$

For the considerable level of 0.05 (*α* = 0.05), the critical value of freedom degrees in the Kwiatkowski–Phillips–Schmidt–Shin (KPSS) test

The test is named after its authors [29] and is used to assess the overall stationarity of the ACE TS:

15 $$ S^{2} \left( l \right)\,\, = \,\,\frac{1}{n}\sum\limits_{t = 1}^{n} {e_{t}^{2} \, + \,} \frac{2}{n}\sum\limits_{s = 1}^{l} {w\left( {s,l} \right)} \frac{1}{n}\sum\limits_{t = s1}^{n} {e_{t} e_{t - s} } $$

where

16 $$ w\left( {s,l} \right)\,\, = \,1\, - \,s\left( {l\,\, + \,\,1} \right) $$

*n* is the number of TS, *e*_*t*_ is the residuals, and *S*_*t*_^*2*^ is the average square of errors between time 1 and *t*. The statistic used for the "level" and "trend" stationarity tests is given by:

17 $$ \eta_{\tau ,\mu } \, = \,\frac{1}{{n^{2} }}\sum\limits_{t = 1}^{n} {\frac{{S_{t}^{2} }}{{S^{2} \left( l \right)}}} $$

Kwiatkowski et al. [29] calculated the symmetric critical values via Monte Carlo simulation. The probability corresponding to a test statistic higher than 5% indicates stationarity.

### Jarque–Bera (JB) test

The *JB* test [30] is applied to measure the the goodness of fit and the test statistic is expressed as follows:

18 $$ JB\, = \,n\left( {\frac{{S_{k}^{2} }}{6}\, + \,\frac{{\left( {K_{u} \, - \,3} \right)^{2} }}{24}} \right) $$

where *K*_*u*_ is kurtosis, *S*_*k*_ is skewness and *JB* is a chi-square distribution with two degrees of freedom that can be used to assume the data is normal.

## Stationarizing methods

### Trend analysis

In case a significant trend term exists in the TS as detected in the *MK*, seasonal Mann–Kendall (*SMK*) or autocorrelation function (ACF) plot, a trend analysis is the best way to remove or reduce its impact on TS. Then, a linear line is fitted to the TS and is subtracted from the TS values; remaining is a detrended TS.

### Differencing

One of the most widely employed methods of stationarizing TS is differencing. This method eliminates correlations in TS. The non-seasonal differencing method, which is the subtraction of each value from the previous one, removes the trend in variances and jumps. The equation is as follows:

19 $$ {\text{Differenced TS }}\left( t \right)\,\, = \,\,{\text{MED}}\left( t \right)\, - \, {\text{MED}}\left( {t \, - \, {1}} \right) $$

where MED(*t*) represents a studied TS, in this case ACE, recorded at time *t*.

### Stochastic modeling

The auto-regressive moving average (ARMA) and auto-regressive integrated moving average (ARIMA) models are the two most conventional methods of the stochastic approach. The difference between these models is in the data differencing method of the ARIMA model, which makes it suitable for non-stationary TS. The equation for ARIMA(*p*, *d*, *q*) is as follows [31]:

20 $$ \varphi \left( I \right)\,\,\left( {1\, - \,I} \right)^{d} MED\left( t \right)\, = \,\theta \left( I \right)\,\varepsilon \left( t \right) $$

21 $$ \varphi \left( I \right)\, = \,\left( {1\, - \,\varphi_{1} I\, - \,\varphi_{2} I^{2} \, - \,\,\varphi_{3} I^{3} \, -  \, \cdots  - \varphi_{p} I^{p} } \right) $$

22 $$ \theta \left( I \right)\, = \,\left( {1\, - \,\theta_{1} I\, - \,\theta_{2} I^{2} \,\, - \,\theta_{3} I^{3} \, - \,\, \cdots \, - - \theta_{p} I^{p} } \right) $$

where *φ* is the autoregressive (AR) process, *θ* the moving average (MA) parameter, *ε*(*t*) the residual, *d* the non-seasonal differencing, and *p* and *q* the AR and MA orders of the model parameters respectively. The value of these orders is determined through autocorrelation function (ACF) and partial autocorrelation (PACF) diagrams [31], *I* the differencing operator, and (1 *− I*)^*d*^ the *d*th non-seasonal differencing. In the ARMA model, *d* is equal to 0 and it does not have the differencing operator.

As it is crucial to investigate the structure of the TS being studied prior to modeling, certain tests and preprocessing methods were initially applied to prepare the data for stochastic modeling. After separation of the dataset into training and testing samples, the existence of deterministic terms in the TS should be examined. For this purpose, *MW*, *MK* and Fisher tests are employed to check the existence of Jump, Trend and Period (respectively).

If the results of these tests show no deterministic terms, the stationary TS must be checked. Otherwise, any deterministic terms should be eliminated. The *KPSS* test is applied to check the stationary TS. If the result of this test does not confirm the stationary TS, Trend analysis and differencing is applied and the *KPSS* test is applied again to check the stationary TS. After ensuring that the TS is stationary, the TS normality is evaluated using the *JB* test. After making sure that the TS is stationary and normal, the preprocessing is finished and stochastic modeling is initiated. Initially, depending on the type of problem, it is determined whether the problem is seasonal or not. Then, the range of seasonal and non-seasonal parameters related to auto regressive (AR) and moving average (MA) terms, as well as a constant term, are determined using ACF and PACF diagrams. The ACF and PACF diagrams only determine the most important lags, not the optimum ones.

**Fig. 2:**
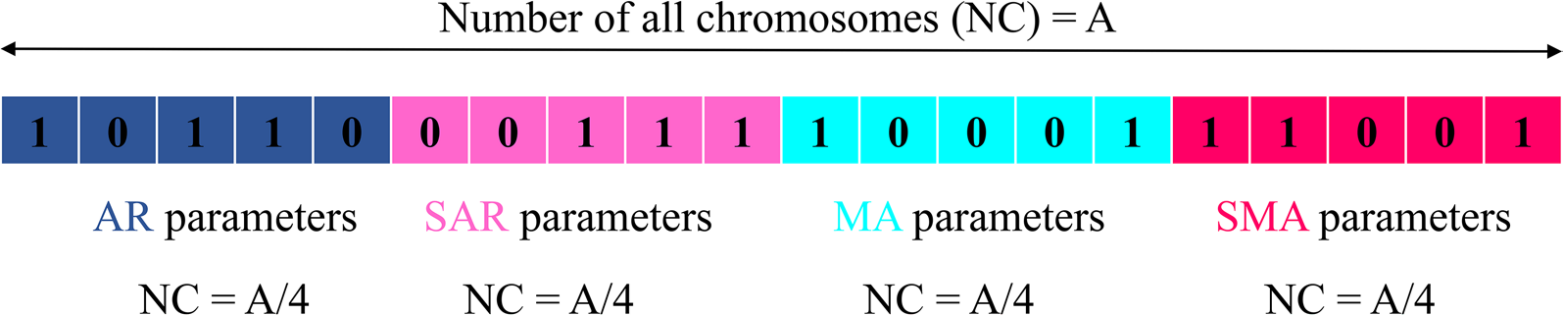
An example of the integrated stochastic model with genetic algorithm. AR, auto regressive; SAR, seasonal auto regressive; MA, moving average; SMA, seasonal moving average

It may be possible to obtain the optimal model; it does not require the use of all the parameters specified by these two diagrams. The first way to obtain the optimum combination is to examine all the compounds resulting from the defined domains for the stochastic model parameters (i.e. 2^*p*(max)+*q*(max)^ − 1 models for an ARMA model). Doing this is very time-consuming as one has to examine all the comparisons and compare them, and the results in many models should be examined as well. Therefore, integrating a stochastic model with the continuous genetic algorithm (CGA) is used in the current study. Indeed, the optimal values of the seasonal MA and AR parameters are determined through an evolutionary process. Then, the residual independence of the proposed model is evaluated using the Ljung-Box test. Finally, the performance of the model is appraised using test data. Considering the maximum number of ARMA, seasonal auto regressive (SAR) and seasonal moving average (SMA) as 5, an example of the optimum achieved solution by ARIMA-CGA is provided in Fig. 2.

**Fig. 3:**
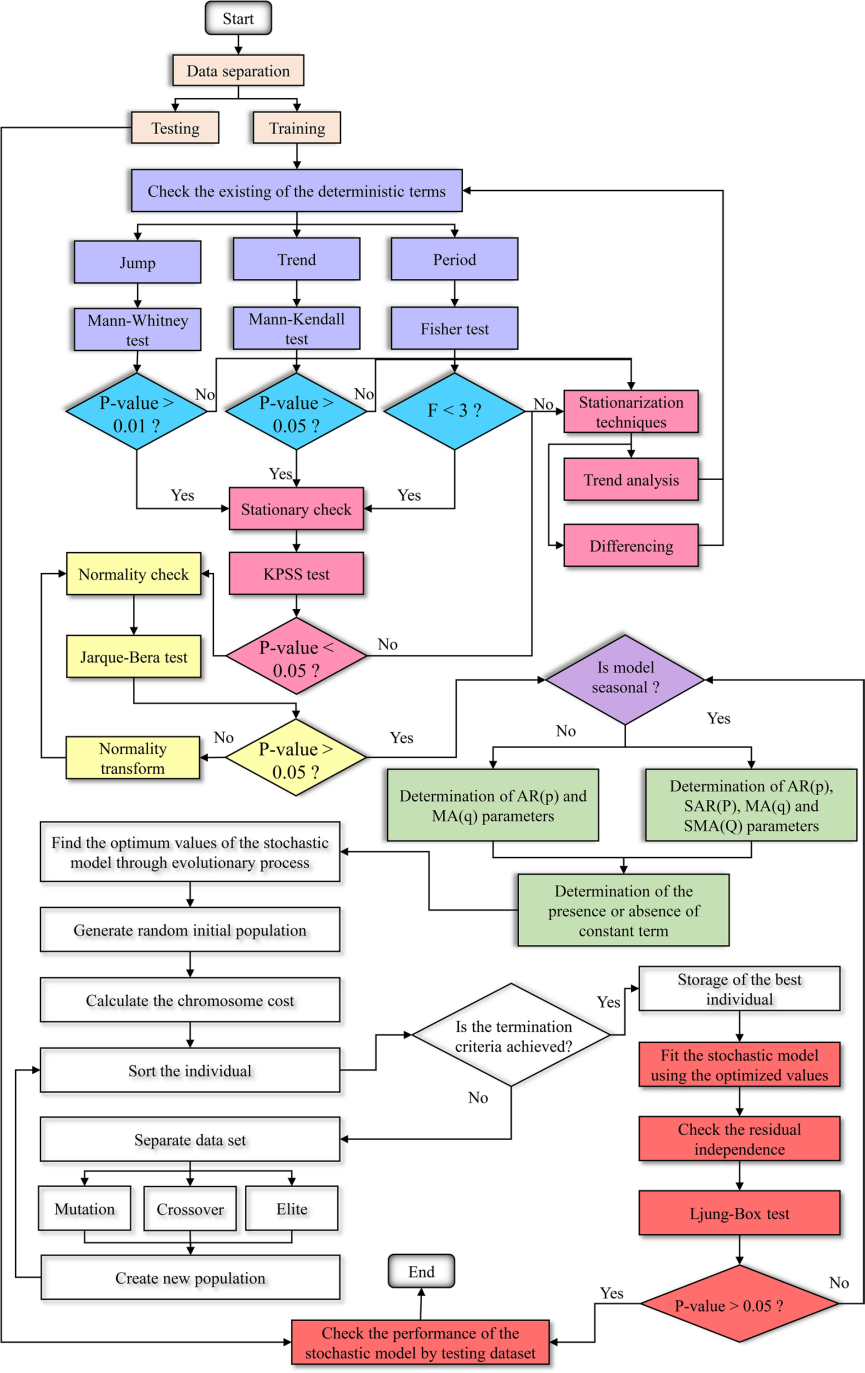
Flowchart of the preprocessing-based stochastic model. KPSS, Kwiatkowski–Phillips–Schmidt–Shin test; F, Fisher test; AR(p), auto regressive model; SAR(P), seasonal auto regressive model; MA(q), moving average model; SMA(Q), seasonal moving average model

The objective function of the CGA is defined, in which all possible combinations are considered and the corrected Akaike information criterion (*AICC*) (Eq. 23) is employed to find the optimum model in terms of accuracy and simplicity simultaneously. The first term of the *AICC* indicates the accuracy of the model while the second one considers the complexity of the model.

23 $$ AICC\, = \,N\, \times \,Ln\left( {MSE} \right)\, + \,2\, \times \,Comp.\, + \,\frac{2 \times Comp.(Comp.\, + \,1)}{{N\, - \,Comp.\, - \,1}} $$

where *N* is the number of samples, *MSE* is the mean square error and *Comp*. is the complexity of the model. The Comp. is the summation of stochastic models (*p*, *q*, *P*, *Q*) and constant term if it exists. The *MSE* is calculated as:

24 $$ MSE\, = \,\,\,\,\frac{{\sum\nolimits_{i = 1}^{N} {(MED_{obs,i} \, - \,MED_{P,i} )^{2} } }}{N} $$

where *MED*_*obs*,i_ and *MED*_*p*,i_ are the *ith* value of the observed and predicted value (respectively). The flowchart of the preprocessing based stochastic model is presented in Fig. 3.

### Generalized structure of group method of data handling (GMDH)

GMDH is a self-organized approach that gradually produces more complex models when evaluating the performance of the input and output datasets [32]. In this approach, the relationship between the input and output variables is expressed by the Volterra Series, which is similar to the Kolmogrov–Gabor polynomial:

25 $$ y\, = \,a_{0} \, + \,\sum\limits_{i = 1}^{N} {a_{i} x_{i} } \,\, + \,\,\sum\limits_{i = 1}^{N} {\sum\limits_{j = 1}^{N} {a_{ij} x_{i} x_{j} } } \, + \,\sum\limits_{i = 1}^{N} {\sum\limits_{j = 1}^{N} {\sum\limits_{k = 1}^{N} {a_{ijk} x_{i} x_{j} x_{k} } \, + \, \cdots } } $$

where *y* is the output variable, *A* = (*a*_*0*_, *a*_*1*_, *…*, *a*_*m*_) is the weights vector and *X* = (*x*_*1*_, …, *x*_*N*_) is the input variables vector. The GMDH model has been developed based on heuristic self-organization to overcome the complexities of multidimensional problems. This method first considers different neurons with two input variables and then specifies a threshold value to determine the variables that cannot reach the performance level. This procedure is a self-organizing algorithm.

The main purpose of the GMDH network is to construct a function in a feed-forward network on the basis of a second-degree transfer function. The number of layers and neurons within the hidden layers, the effective input variables and the optimal model structure are automatically determined with this algorithm.

In order to model using the GMDH algorithm, the entire dataset should first be divided into training and testing categories. After segmenting the data, it creates neurons with two inputs. Given that each neuron has only two inputs, all possible combinations for a model with *n* input vectors are as:

26 $$ NAPC\, = \,\left( \begin{gathered} n \hfill \\ 2 \hfill \\ \end{gathered} \right)\, = \,\frac{n(n\, - \,1)}{2} $$

where *NAPC* is the number of all possible combinations and *n* is the number of input vectors.

According to the quadratic regression polynomial function, all neurons have two inputs and one output with the same structure, and each neuron with five weights (*a*_*1*_, *a*_*2*_, *a*_*3*_, *a*_*4*_, *a*_*5*_) and one bias (*a*_*0*_) executes the processing between the inputs (*x*_*i*_, *x*_*j*_) and output data as follows:

27 $$ \overset{\lower0.5em\hbox{$\smash{\scriptscriptstyle\frown}$}}{y} \, = \,\overset{\lower0.5em\hbox{$\smash{\scriptscriptstyle\frown}$}}{f} (x_{i} ,x_{j} )\, = \,a_{0} \, + \,a_{{1}} x_{i} \, + \,a_{{2}} x_{j} \, + \,a_{{3}} x_{i}^{2} \, + \,a_{{4}} x_{j}^{2} + \,a_{{5}} x_{i} x_{j} $$

The unknown coefficients (*a*_*0*_, *a*_*1*_, *a*_*2*_, *a*_*3*_, *a*_*4*_, *a*_*5*_) are obtained by ordinary least squares. The performance of all neural network methods is heavily influenced by the chosen parameters. The unknown coefficients are calculated through a least squares solution as follows:

28 $$ A_{i} \, = \,(x_{i}^{T} x_{i} )^{ - 1} x_{i} Y $$

where *A* = {*a*_*0*_, *a*_*1*_, *a*_*2*_, *a*_*3*_, *a*_*4*_, *a*_*5*_} is the unknown coefficients vector, *Y* = {*y*_*1*_, …, *y*_*N*_}^*T*^ is the output vector and x is the input variable vector.

The *AICC* criterion (Eq. 23) is applied to determine the optimal network structure and select the neurons describing the target parameter. The *Comp*. in this equation for the GMDH model is defined as follows:

29 $$ Comp.\, = \,NL\, + \,NTN $$

where *Comp*. is the complexity, *NL* is the number of layers and *NTN* is the number of total neurons.

The performance of classical GMDH in the modeling of nonlinear problems has been demonstrated in various studies [33–36]. However, along with its advantages, it possesses the following limitations: (i) second-order polynomials, (ii) only two inputs for each neuron, (iii) inputs of each neuron can only be selected from the adjacent layer [37, 38]. In complex nonlinear problems, the necessity of using second-order polynomials may impede an acceptable result. In addition, considering only two inputs per neuron and using adjacent layer neurons would result in a significant increase in the number of neurons (NN) [39].

In the current study, a new scheme of GMDH as a GS-GMDH is employed and encoded in the MATLAB environment. The developed model removes all the mentioned disadvantages, so that each neuron can connect to two or three neurons at a time, taken from adjacent or non-adjacent layers. In addition, the order of polynomials can also be two or three. Similar to classical GMDH, the best structure is chosen based on the *AICC* index. According to the provided description, the developed GS-GMDH can offer four modes: (1) second-order polynomial with two inputs, (2) second-order polynomial with three inputs, (3) third-order polynomial with two inputs, and (4) third-order polynomial with three inputs. The first mode is classical GMDH.

**Fig. 4:**
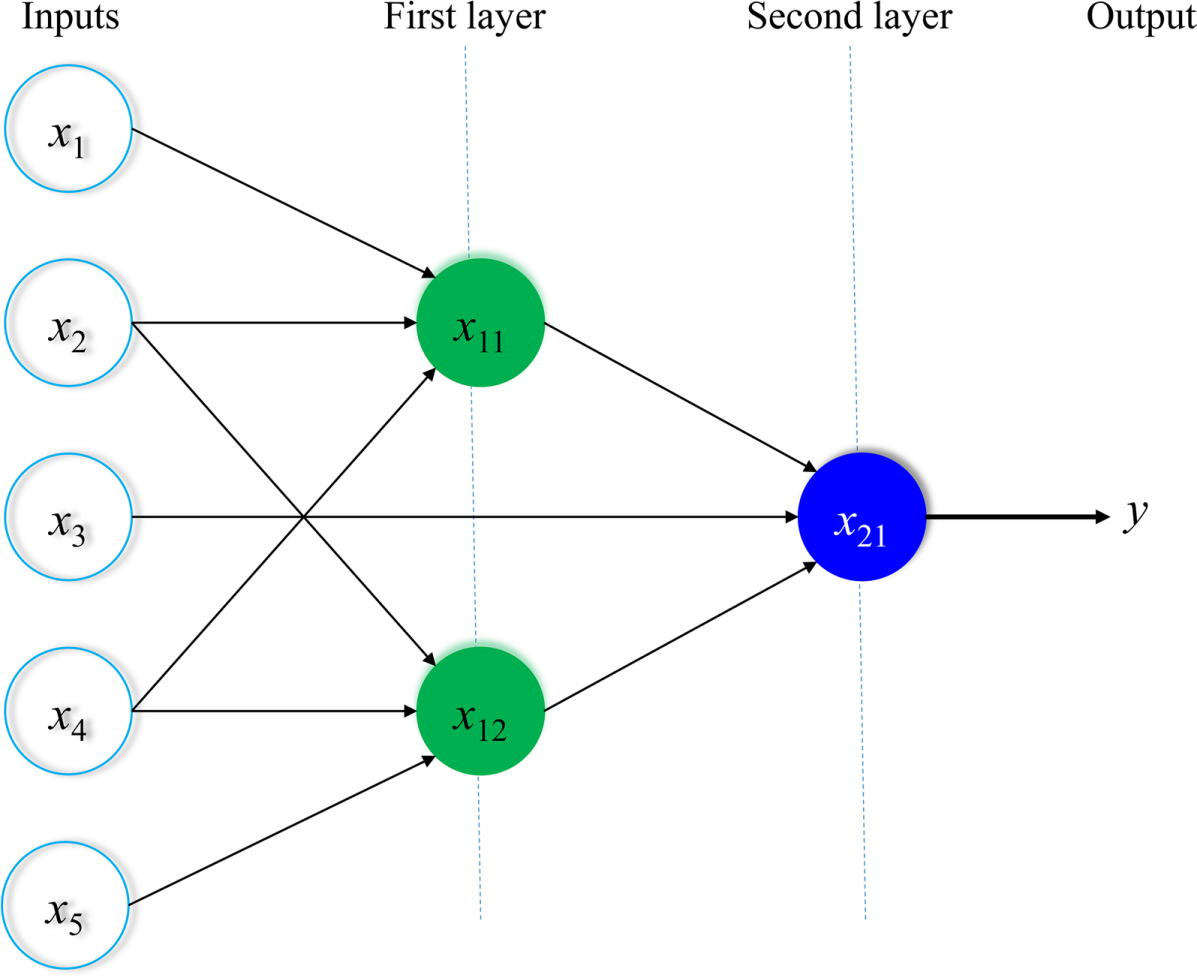
An example of the developed generalized structure group method of data handling (GS-GMDH) for a model with five inputs and one output. × 1, ×2, ×3, ×4, ×5, input parameters; ×11, × 2, neurons in first layer; ×21, neuron in second layer; y, output

Figure 4 indicates an example of the developed GS-GMDH for a model with five inputs and one output. In this figure, 3 different neurons (*x*_11_, *x*_12,_ *x*_21_) are presented to provide an equation to estimate the target parameter (*y*). The two neurons *x*_11_ and *x*_21_ have three inputs, which are the inputs of the desired problem. The *x*_21_ neuron, which is the output of the problem, has three inputs similar to the two previous neurons (*x*_11_ and *x*_21_), except that it uses the non-adjacent layer neurons (*x*_13_) in addition to the adjacent layer neurons (*x*_11_ and *x*_21_).

**Fig. 5:**
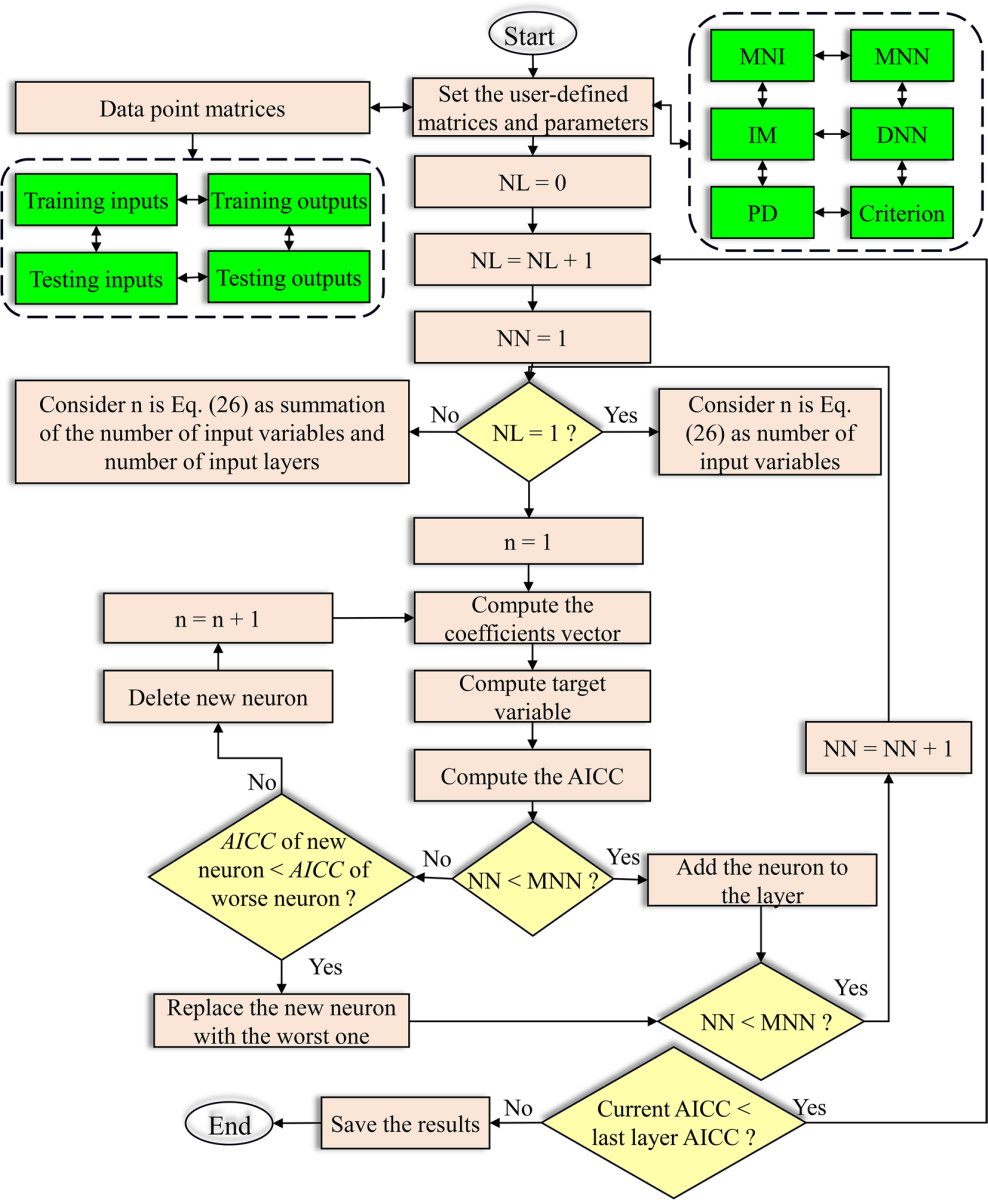
The flow chart of the developed generalized structure group method of data handling (GS-GMDH). MNI, maximum number of inputs; MNN, maximum number of neurons; IM, inputs more; DNN, decrease number of neurons; PD, polynomial degree; NL, number of layer; NN, number of neuron; n, number of input vectors; AICC, Akaike information criterion

The GS-GMDH was used in this study to achieve the most precise results in forecasting the studied TS, which we abbreviated as MED data. GS-GMDH is superior to the former method, GMDH, due to the random structure of neurons that is encoded in the genotype string that results in using all neurons from previous layers in subsequent layers. In addition, GS-GMDH facilitates finding the minimized training and prediction errors separately, preventing model overtraining. The flow chart of the developed GS-GMDH model is presented in Fig. 5.

Before starting the modeling using the GS-GMDH method, some parameters must first be determined. The first parameter is the Maximum Number of Inputs (MNI) that determines the maximum number of inputs for individual neurons. It could be two or three. If set to three, both two and three inputs are tried. Inputs More (IM) is the other one that should be determined before starting modeling. It could be zero or one. If set to zero, the inputs of each neuron are considered only for previous layer while if IM is set to one, this results in taking input from the non-adjacent layers also. The Maximum Number of Neurons (MNN) is equal to the number of input variables, while it could be twice that number for complex problems. The polynomial degree (PD) could be considered to be two or three. If set to three, both two and three are allowed.

### Combining linear and nonlinear models (data-driven method)

**Fig. 6:**
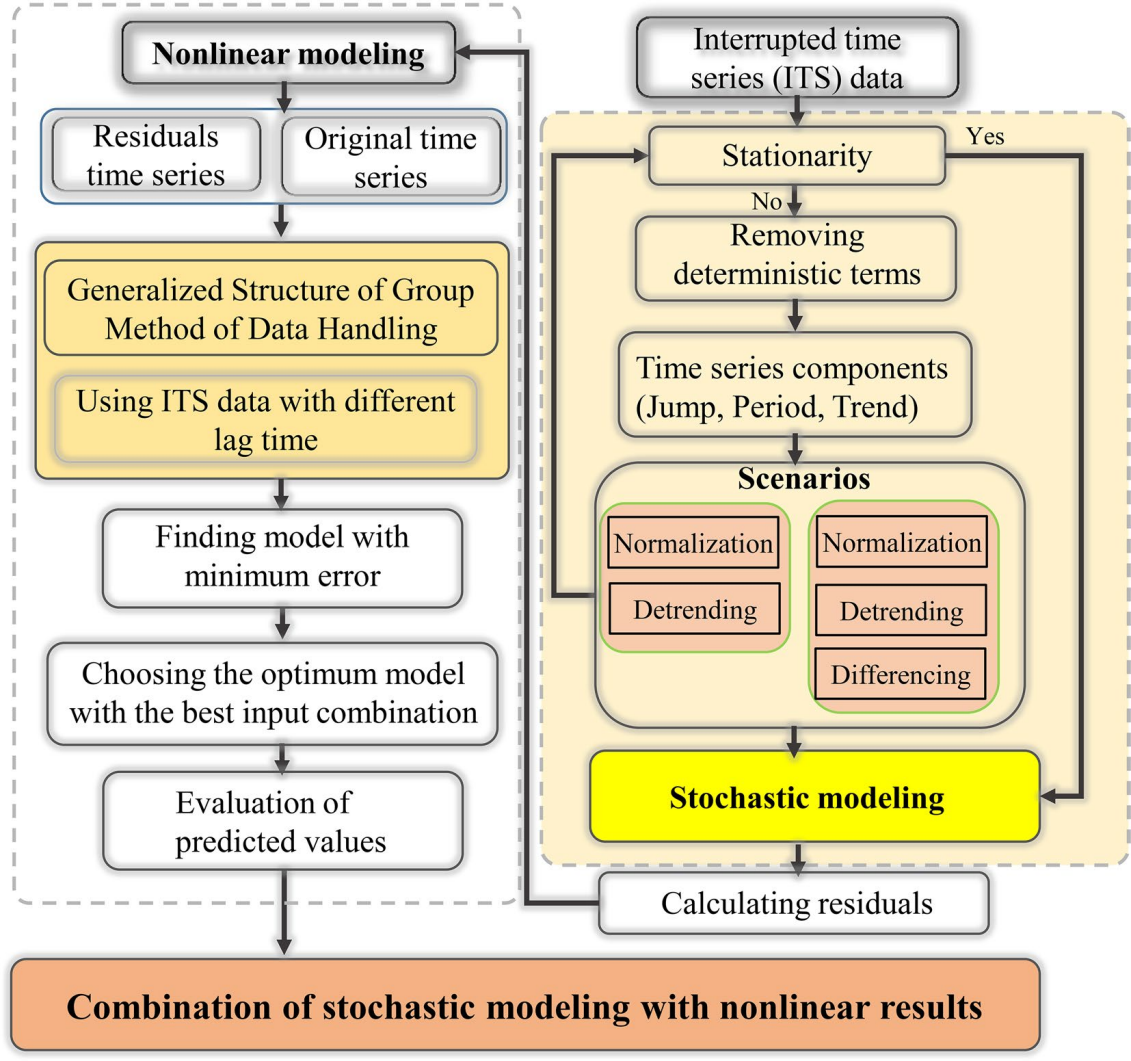
Flowchart of interrupted time series modeling through a continuous nonlinear approach

ITS consists of stochastic and deterministic components. Thus, by using appropriate data preprocessing methods, it is possible to reduce the problematic effects of deterministic components in the modeling process. The proposed methodology is based on a continuous modeling process. This data-driven method is based on preprocessing to identify linear and nonlinear components of ITS, verification of the validity of decomposed data, and the decomposed model. In the studied case (6), the ACE TS fluctuates greatly. The outcomes of the single stochastic and neural network modeling approaches are relatively weak. Hence, as a third approach, the ACE TS is modeled with a combined stochastic-neural network model. Stochastic models perform efficiently, while TS are linear and do not contain deterministic terms that are responsible for nonlinearity. AI methods, on the other hand, allow the modeling of TS with nonlinear components. The TS, however, is not purely linear or nonlinear; both components are present simultaneously; the integration of which sometimes produces complex structures in the TS. In such cases, the use of single stochastic or nonlinear methods might be improved by a combined model. Combining stochastic models with AI methods is one of the most effective methods of modeling TS with complex structures. As shown in Fig. 6, the residuals of the stochastic models were used as a new TS in GS-GMDH modeling, such that the features of both modeling approaches were utilized.

### Verification indices to evaluate models

To verify the accuracy of modeling performed in the TS MED forecasting, the correlation coefficient (*R*), scatter index (*SI*), mean absolute percentage error (*MAPE*), root mean squared relative error (*RMSRE*) and performance index (*ρ*) are used. In addition to these indices, the corrected *AICC* and Nash–Sutcliffe model efficiency (*E*_*N-S*_) based on comparing the model's simplicity with the goodness-of-fit and amount of deviation from the mean value [40] are used. The *AICC* index is used to find the best models in each TS modeling, and the lower the index value is the simpler the model. The *E*_*N-S*_ index ranges from -∞ to 1, and the closer the index is to one, the more accurate the model.

30 $$ R\, = \,\frac{{\sum\limits_{i = 1}^{N} {\left( {MED_{obs,i} \, - \,\overline{MED}_{obs,i} } \right)\left( {MED_{pred,i} \, - \overline{\,MED}_{pred,i} } \right)} }}{{\sqrt {\sum\limits_{i = 1}^{N} {\left( {MED_{obs,i} \, - \,\overline{MED}_{obs,i} } \right)^{2} } \sum\limits_{i = 1}^{N} {\left( {MED_{pred,i} \, - \,\overline{MED}_{pred,i} } \right)^{2} } } }} $$

31 $$ SI\, = \,\frac{{\sqrt {\frac{1}{N}\sum\limits_{i = 1}^{N} {\left( {MED_{obs,i} \, - \,MED_{pred,i} } \right)^{2} } } }}{{\overline{MED}_{obs} }} $$

32 $$ MAPE = \,\frac{100}{N}\left( {\frac{{\left| {MED_{obs,i} \, - \,MED_{pred,i} } \right|}}{{MED_{obs,i} }}} \right) $$

33 $$ RMSE\, = \,\sqrt {\frac{1}{N}\sum\limits_{i = 1}^{N} {\left( {MED_{obs,i} \, - \,MED_{pred,i} } \right)^{2} } } $$

34 $$ \rho \, = \,\frac{{\left( {\sqrt {\frac{1}{N}\sum\limits_{i = 1}^{N} {\left( {MED_{obs,i} \, - \,MED_{pred,i} } \right)^{2} } } /\sum\limits_{i = 1}^{N} {\left( {MED_{obs,i} } \right)} } \right)}}{1\, + \,R} $$

35 $$ E_{N - S} \,\,\, = \,\,\left[ {1 - \frac{{\sum\limits_{i = 1}^{N} {\left( {MED_{obs,i} - MED_{pred,i} } \right)^{2} } }}{{\sum\limits_{i = 1}^{N} {\left( {MED_{obs,i} - \overline{MED}_{pred,i} } \right)^{2} } }}} \right] $$

36 $$ AICC\, = \,\frac{{2kN\, + \,\left( {N\ln \left( {\sigma_{\varepsilon }^{2} } \right)\left( {N\, - \,k\, - \,1} \right)} \right)}}{N\, - \,k\, - \,1} $$

where *k* is the number of parameters, *N* is the number of samples, *σ*_*ε*_^*2*^ is the residuals’ standard deviation, *E*_*N-S*_ is the Nash–Sutcliffe test statistic, and *MED*_*obs,i*_ and *MED*_*pred,i*_ are the ith value of actual data and forecasted MED, respectively.

The Ljung-Box test is used to check the independence of the residuals of the modeled TS [41]. The test statistic is calculated as follows:

37 $$ Q_{Ljung - Box} \, = \,\left( {N^{2} \, + \,2N} \right)\sum\limits_{h = 1}^{m} {\frac{{r_{h} }}{N\, - \,1}} $$

where *N* is the number of samples, *r*_*h*_ is the residual coefficient of the auto regression (*ε*_*t*_) in lag *h*, and the value of *m* is equal to *ln(N)*. If the probability corresponding to the Ljung-Box test statistic in the χ2 distribution is higher than the α-level (in this case *P*_*Q*_ > α = 0.05), the residual series is white noise and the model is adequate.

## Results

### Preprocessing tests

**Fig. 7:**
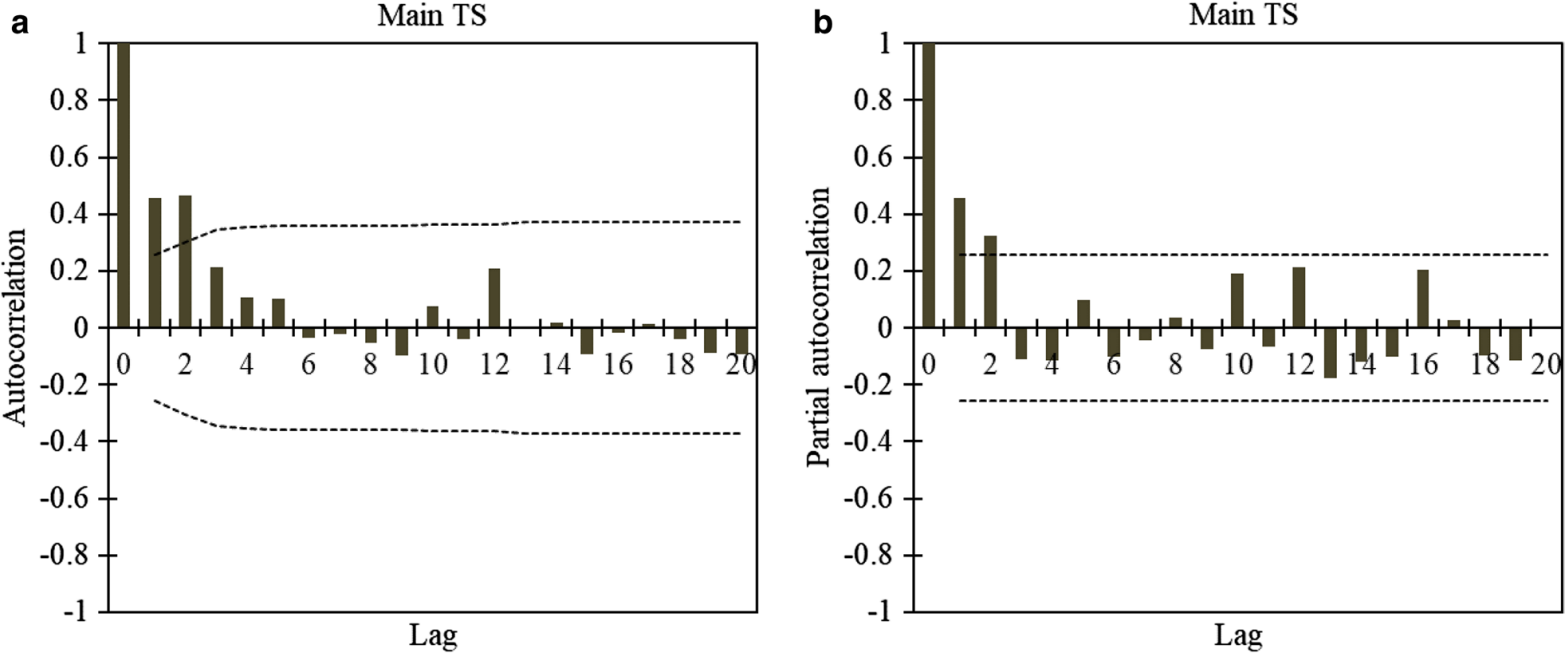
Standardized rate of acute coronary events (ACEs) time series: **a** autocorrelation; **b** partial autocorrelation. Lag indicates the time period

The values of the *JB* test show that the desired TS is distributed normally (*p*_*JB*_ = 66.29 > 0.05). Figure 7 indicates the ACF and the PACF of the main TS (standardized rate of ACEs TS), and data showed that there is a correlation up to three non-seasonal lags (the time period). Since the values of ACF are rapidly damped and are within the limit boundaries, there is no significant period or trend in the TS. However, to ensure this, the existence of deterministic terms and stationarity of the main TS was also evaluated using quantitative tests.

Table 1 provides the results of the quantitative test to evaluate the existence of deterministic terms, stationarity and normality of the main series, and detrended and differenced TS. The results of the non-seasonal and seasonal *MK* tests show that the p-values of *MK* and seasonal *MK* are 0.02 and 0.24 respectively. Therefore, the ACE TS has a non-seasonal trend (*p*_*MK*_ = 0.02 is less than critical value, 0.05). Hence, the trend must be removed. Moreover, the p-value of the Fisher test indicates that the TS has a period (*p*_*Fisher*_ = 5.85) greater than the critical value 3. According to the Fisher test, the severity of the period is not very high as the value is close to critical, then minor. Moreover, the *MW* test proves there is no jump in the TS (*p*_*MW*_ = 2.78, higher than the acceptable value 0.05). The *KPSS* test also indicates the TS is non-stationary (*p*_*KPSS*_ = 0.19, higher than the acceptable value 0.05), which is because of the trend and period detected in the TS.

**Table 1:** Evaluation of the presence of deterministic terms; stationarity and normality of the standardized rate of acute coronary events (ACEs) time series; and detrended and differenced time series

Tests Time series	Trend		Jump	Period	Stationarity	Normality
	Mann–Kendall	Seasonal Mann–Kendall	Mann–Whitney	Fisher	Kwiatkowski–Phillips–Schmidt–Shin	Jarque–Bera
Main time series	0.02	0.24	2.78	5.85	0.19	66.29
Detrended time series	100	37.75	46.77	1.14E + 03	44.55	53.06
Differenced time series	81.96	28.07	97.54	1.25E + 04	98.48	54.48

Fisher critical value: 3. Fisher < 3 means the absence of a period, other tests: critical value 0.05, acceptable value > 0.05

To remove the deterministic terms, two scenarios are defined: de-trending and stationarizing the ACE TS by differencing before stochastic modeling. The linear trend line is obtained as follows: trend line = 0.4792 × *t* + 201.72.

**Fig. 8:**
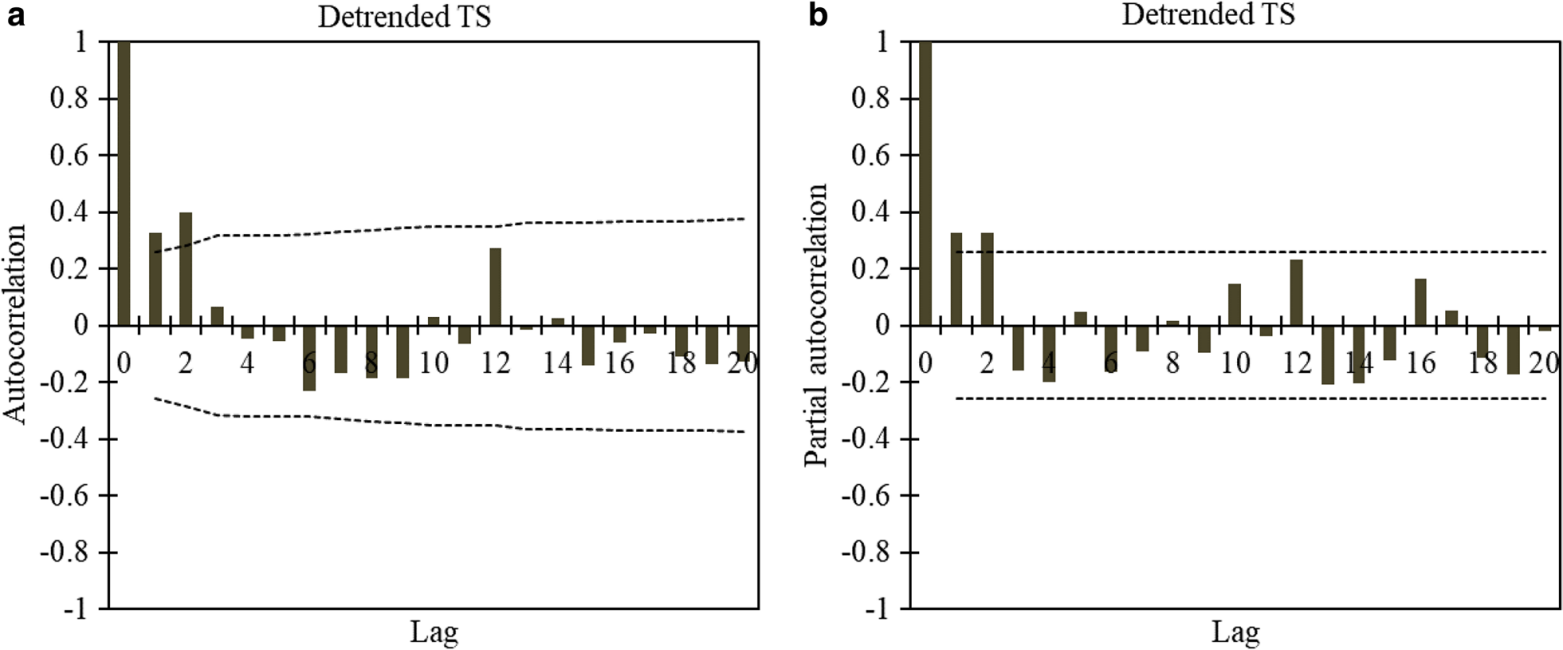
Detrended time series: **a** autocorrelation, **b** partial autocorrelation. Lag indicates the time period

After eliminating the linear trend from the main TS, all of the deterministic factors are removed. Indeed, the detrended TS is stationary with no deterministic term. Similar to the main TS, this has a normal distribution. Consequently, the detrended TS is modeled with the ARMA. To find the parameters of the ARMA model, ACF and PACF diagrams are employed. As shown in Fig. 8, it is obvious there is still a correlation to three non-seasonal lags. Therefore, p and q in ARMA(*p*,*q*) are considered as *p*,*q* = {0,1,2,3}. For the purpose of determining more accurate and simpler models, the value of these parameters is considered 10.

**Fig. 9:**
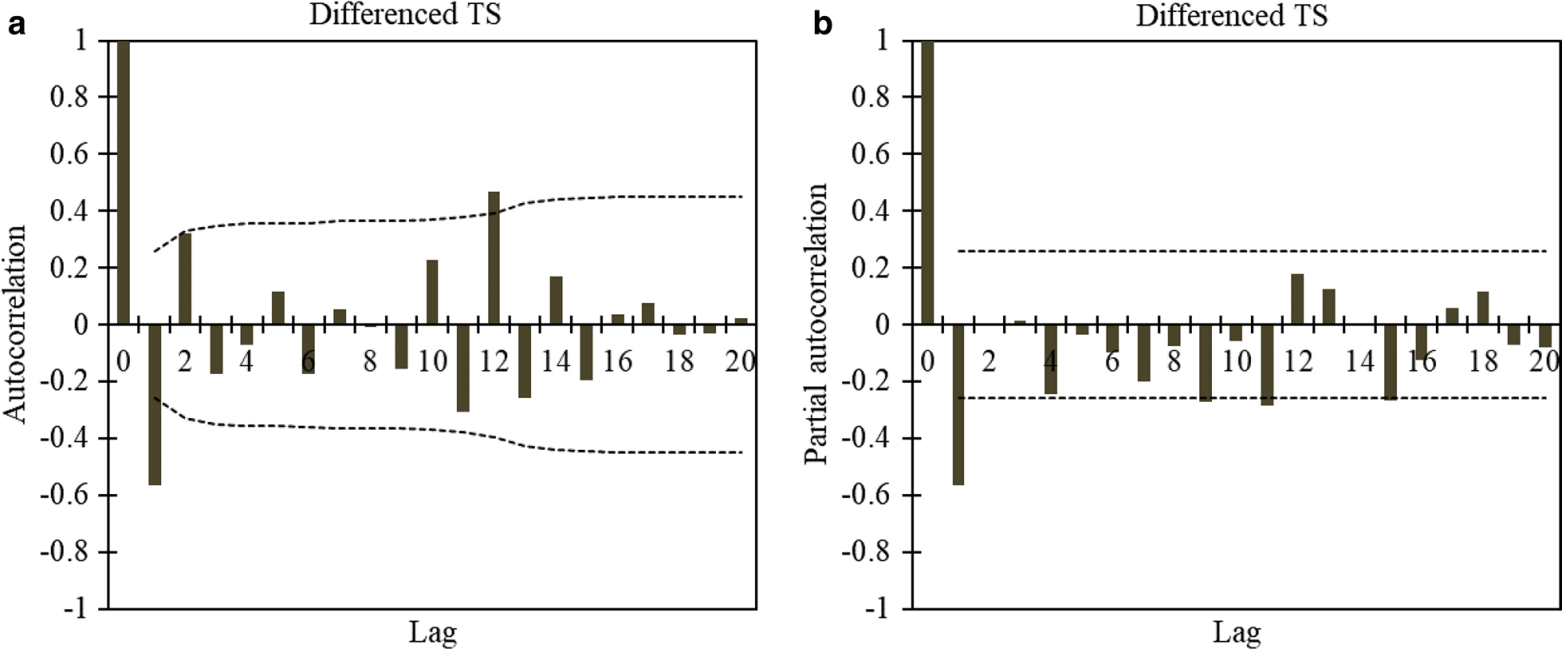
Differenced time series: **a** autocorrelation, **b** partial autocorrelation. Lag indicates the time period

In the second scenario, differencing the main TS is proposed (differenced TS) to remove the deterministic terms. The findings in Table 1 in which *MW*, *MK*, *SMK* and *JB* increases are higher than 0.05, as well as the Fisher test higher than 3, indicate the differenced TS results in an increasing of the period in the new TS. Although the Fisher test exhibits growth in periodicity, the stationarity of the differenced TS increases considerably; thus, enabling the modeling of the TS. Furthermore, the differencing method has considerable impact on the correlation of the lags and decreases them markedly. Hence, an ARIMA model could be employed with fewer parameters and subsequently less error. The ACF and PACF of the differenced TS (Fig. 9) indicate that the values of p and q in this state are lower than the ARMA model.

### Stochastic modeling

**Fig. 10:**
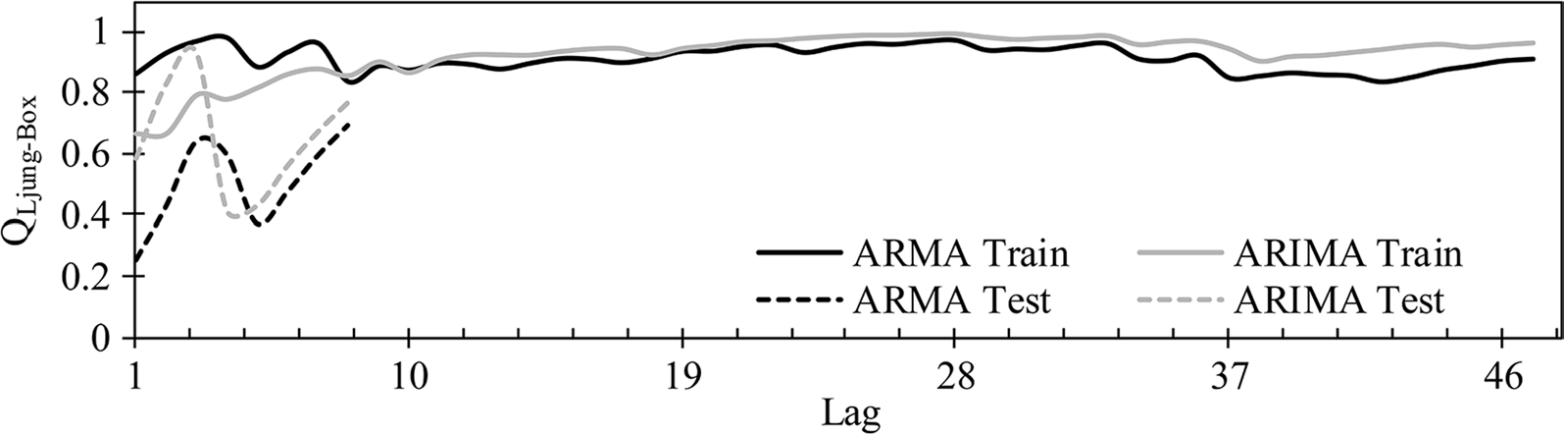
Ljung–Box results of the auto-regressive moving average (ARMA) and auto-regressive integrated moving average (ARIMA) models for both training (train) and testing (test) stages. Lag indicates the time period

**Fig. 11:**
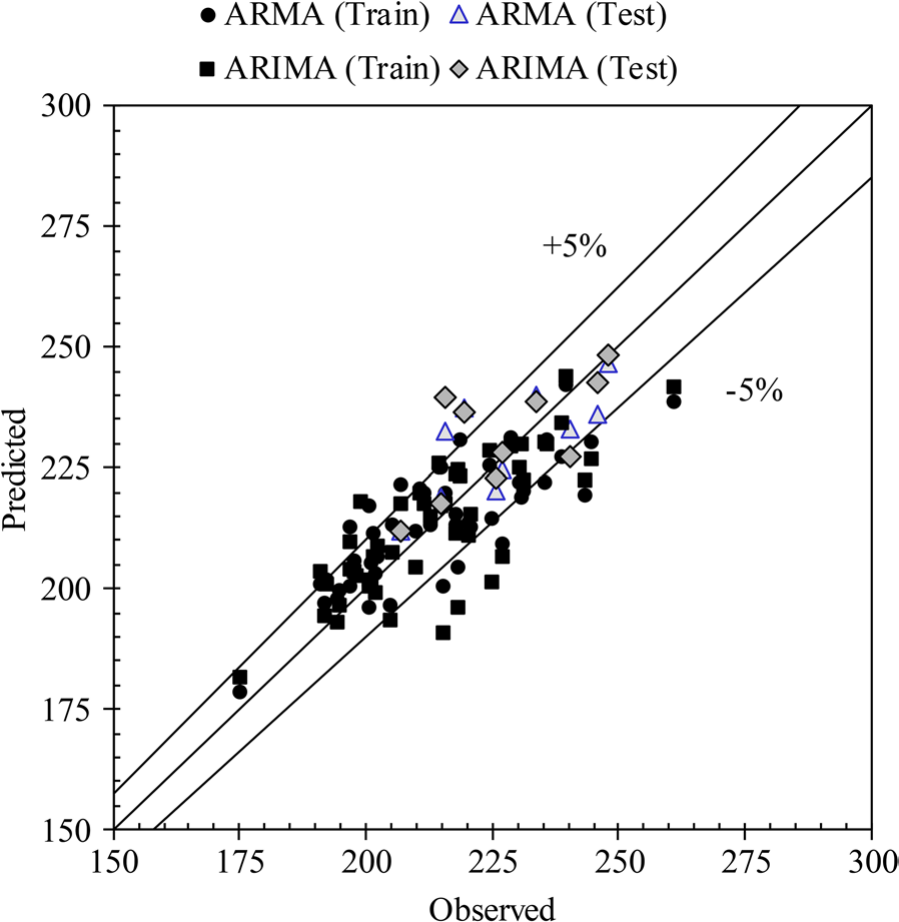
Scatter plot of the auto-regressive moving average (ARMA) and auto-regressive integrated moving average (ARIMA) model predictions of MED data

TS modeling, however, offers numerous combinations of previous lags from which to select the most appropriate TS input combination. Therefore, applying suitable preprocessing should lead to determining and selecting the most effective lag for modeling. According to the ACF plots for both preprocessed TS and test results, a maximum of three parameter orders are required for ARMA modeling and one for ARIMA modeling (Figs. 7, 8, 9). For modeling, the first 50 data were considered for the training stage and the remainder (nine data) for the testing stage. The stochastic-based linear modeling results are presented in Table 2. As the results in this table indicate, both linear models are relatively weak in modeling the ACE TS. The ARMA model outperforms ARIMA and the results are marginally better than ARIMA. The ARMA model with seven non-seasonal auto-regressive parameters and five non-seasonal moving average parameters modeled the ACE TS with *R* = 77.95%, *SI* = 3.46%, *MAPE* = 2.89%, *RMSRE* = 3.54%, *E*_*N-S*_ = 0.66 and *AICC* = −15.84 in testing. The ARIMA model also performed slightly weaker than ARMA with *R* = 73.74%, *SI* = 4.21%, *MAPE* = 2.89%, *RMSRE* = 4.37, *E*_*N-S*_ = 0.50 and *AICC* = −5.55 in testing. The Ljung-Box results for ARIMA and ARMA models are provided in Fig. 10. The test is done for the first 47 lags of the training part and the eight lags of the test part separately (*n*-1 data are considered for testing). It is observed that the residuals of both linear models are independent and the white noise and modeling are adequate and correct. Figure 11 demonstrates scatter plots of both ARMA and ARIMA models in testing and training versus the observed data. According to this figure, the majority of forecasted data are located within 5% intervals.

**Table 2:** Statistical indices for the stochastic-based linear modeling

Superior models			*R*%	*SI*%	*MAPE*%	*RMSRE*%	*E*_*NS*_	*AICC*
ARMA	(7,5)	Train	77.95	4.94	4.16	4.84	0.61	269.04
		Test	81.87	3.46	2.89	3.54	0.66	−15.84
ARIMA	(9,1,4)	Train	78.60	4.89	3.75	4.78	0.61	271.71
		Test	73.74	4.21	2.89	4.37	0.50	−5.55

*ARMA (7,5)* auto-regressive moving average for seven non-seasonal auto-regressive parameters and five non-seasonal moving average parameters, *ARIMA (9,1,4)* auto-regressive integrated moving average for nine non-seasonal auto-regressive parameters, one non-seasonal differencing and four non-seasonal moving average parameters, *R* correlation coefficient, *SI* Scatter index, *MAPE* mean absolute percentage error, *RSMRE* root mean square relative error, *E*_*N-S*_ Nash–Sutcliffe model efficiency, *AICC* Akaike information criterion

### Generalized structure group method of data handling (GS-GMDH)

**Fig. 12:**
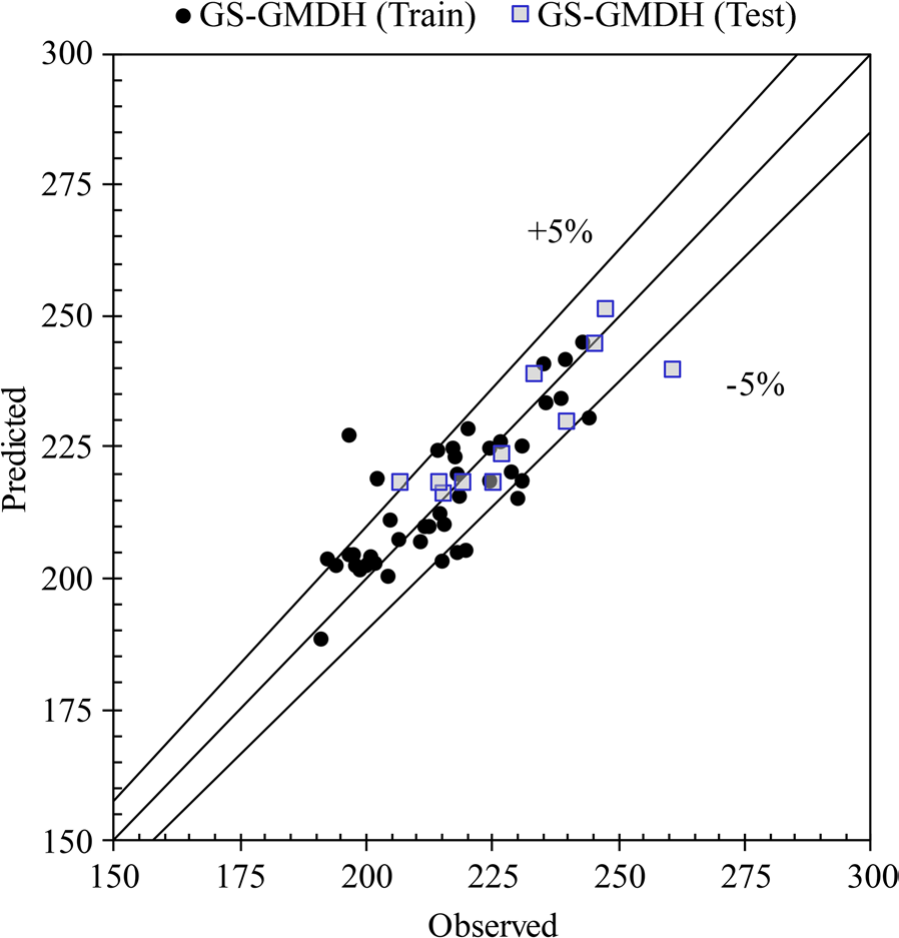
Scatter plot of generalized structure group method of data handling (GS-GMDH) in MED prediction

As mentioned earlier, AI methods are widely utilized for data simulation and forecasting. Each TS consists of two parts: linear and nonlinear. The stochastic models, also known as linear models, are able to model the linear part of the TS; hence, the nonlinearity is removed from the TS prior to modeling with pre-preprocessing methods. Conversely, AI models are known for their ability in modeling the nonlinear part. The neural network applied to the ACE TS under study is the GS-GMDH model, which is enhanced by the genetic algorithm. In this GS-GMDH, it is allowed to randomly apply crossover and mutation for the whole length of the chromosome string. Neurons are used for all layers and by calculating the errors separately; both training and testing sets have low errors. The results of this method are presented in Table 3. According to the results, the model was able to forecast the original TS without preprocessing with *R* = 82.35%, *SI* = 4.22%, *MAPE* = 3.16%, *RMSRE* = 4.25% and *ρ* = 2.33% for the training data and *R* = 89.35%, *SI* = 2.60%, *MAPE* = 2.10%, *RMSRE* = 2.66% and *ρ* = 2.33% for the testing data. As the scatter plot for both training and testing data in Fig. 12 indicates, the majority of forecasted data in the testing period have less than 5% error and are located within the intervals. The AI method employed allowed forecasting of the nonlinearity in the ACE TS very well. Though the GS-GMDH method performed better than the single ARIMA and ARMA models, with a mean growth of 11.63% in correlation, these results are relatively close to the stochastic models. Therefore, a complementary method is required.

**Table 3:** Statistical indices for the developed hybrid model in acute coronary event (ACE) forecasting

Model		*R*%	*SI*%	*MAPE*%	*RMSRE*%	*ρ%*
ARMA–GS-GMDH	Train	88.97	3.64	2.84	3.59	1.9
	Test	91.03	2.52	2.13	2.47	1.29
ARIMA–GS-GMDH	Train	84.93	3.91	2.94	3.97	2.15
	Test	91.91	2.86	2.46	2.99	1.56

*R* correlation coefficient, *SI* scatter index, *MAPE* mean absolute percentage error, *RSMRE* root mean square relative error

### Combined data-driven modeling

As mentioned in previous sections, each model has certain specifications. Stochastic models perform efficiently, while TS are linear and do not contain deterministic terms that are responsible for nonlinearity. AI methods, on the other hand, allow the modeling of TS with nonlinear components. The TS, however, is not purely linear or nonlinear. Both components are present simultaneously, the integration of which sometimes creates complex structures. In such cases, employing single stochastic or nonlinear methods does not provide acceptable results. Therefore, alternative solutions are required to resolve this problem. Hybridizing stochastic models with AI methods is one of the most viable methods of modeling TS with complex structures. In the studied case, the ACE TS fluctuates greatly. The outcomes of the single stochastic and neural network modeling approaches are relatively weak. Thus, as a third approach, the ACE TS is modeled with a combined stochastic-neural network model. The hybrid model results are provided in Table 4.

**Table 4:** Statistical indices for the developed hybrid model in acute coronary event (ACE) forecasting

Model		*R*%	*SI*%	*MAPE*%	*RMSRE*%
ARMA–GS-GMDH	Train	88.97	3.64	2.84	3.59
	Test	91.03	2.52	2.13	2.47
ARIMA–GS-GMDH	Train	84.93	3.91	2.94	3.97
	Test	91.91	2.86	2.46	2.99

*ARMA–GS-GMDH* Auto-regressive moving average–generalized structure group method of data handling, *ARIMA–GS-GMDH* Auto-Regressive Integrated Moving Average–Generalized Structure Group Method of Data Handling, *R* coefficient correlation, *SI* scatter index, *MAPE* mean absolute percentage error, *RSMRE* root mean square relative error, ρ% performance index

**Fig. 13:**
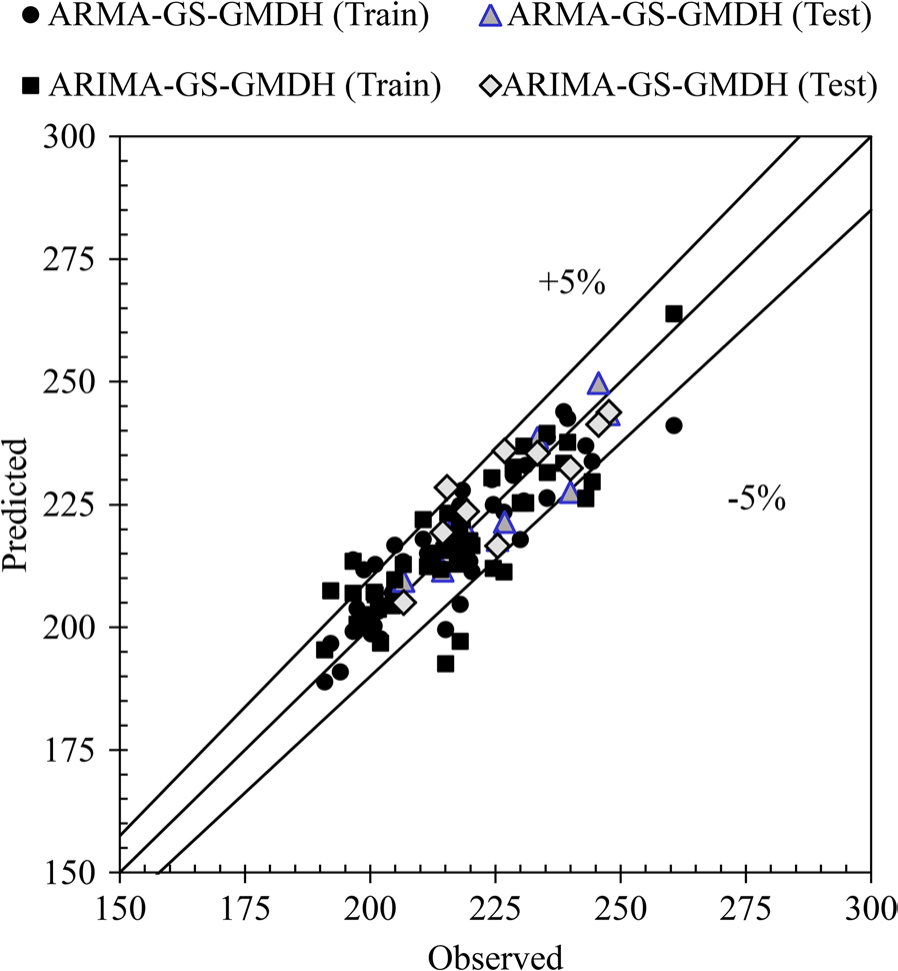
Scatter plot of auto-regressive moving average-generalized structure group method of data handling (ARMA–GS-GMDH) and auto-regressive integrated moving average–generalized structure group method of data handling (ARIMA–GS-GMDH) in acute coronary event (ACE) prediction

**Fig. 14:**
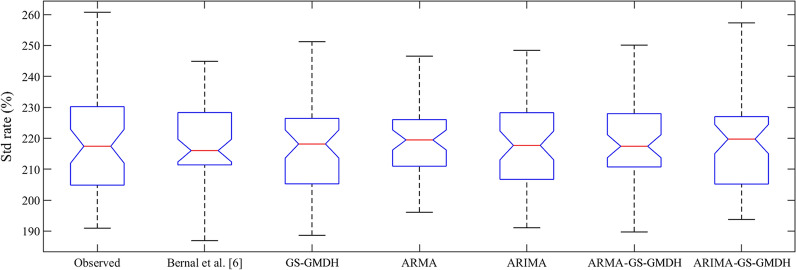
Box plot of all models versus observed data. GS-GMDH, generalized structure group method of data handling; ARMA, auto-regressive moving average; ARIMA, auto-regressive integrated moving average; ARMA–GS-GMDH, auto-regressive moving average–generalized structure group method of data handling; ARIMA–GS-GMDH, auto-regressive integrated moving average–generalized structure group method of data handling; Std rate standardized rate of acute coronary events (ACEs) [6]

**Fig. 15:**
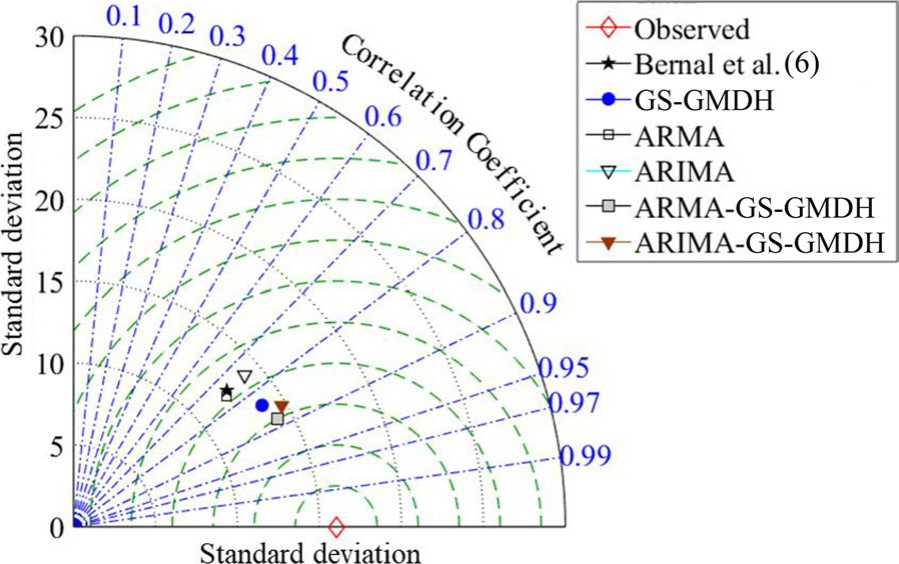
Taylor graph for checking the performance of the linear, nonlinear and hybrid models in predicting acute coronary events (ACEs). GS-GMDH, generalized structure group method of data handling; ARMA, auto-regressive moving average; ARIMA, auto-regressive integrated moving average; ARMA–GS-GMDH, auto-regressive moving average–generalized structure group method of data handling; ARIMA–GS-GMDH, auto-regressive integrated moving average–generalized structure group method of data handling [6]

It is apparent from the information supplied in Table 4 that the correlation between the modeled and observed data is rising. The *R* exhibited an average of 17.74% enhancement from the single stochastic model and 2.05% from the single GS-GMDH model. Although the results are slightly better than the single GS-GMDH model, model accuracy improved and in fact, the errors are about half those of the linear model. The ARMA-GS-GMDH model with *R* = 91.03%, *SI* = 2.52%, *MAPE* = 2.13%, *RMSRE* = 2.47% and *ρ* = 1.29% outperformed the ARIMA–GS-GMDH model with *R* = 91.91%, *SI* = 2.86%, *RMSRE* = 2.99% and *ρ* = 1.56% as well as all other models. Figure 13 demonstrates the scatter plot of the hybrid modeling results, where almost all forecasted data are within the  ± 5% error interval. Figures 14 and 15 provide a good comparison between the observed MED data and the models. The box plot (Fig. 4a) shows that the hybrid model forecasted the interquartile area, mean and median of the data better than other models. However, the maximum and minimum predictions varied between the models (Fig. 4b). The superiority of the ARIMA–GS-GMDH model is demonstrated by the model’s maximum, minimum and interquartile areas, which are much closer to the observed data than all other models, especially the regression model used in the Bernal et al. [6] study.

**Fig. 16:**
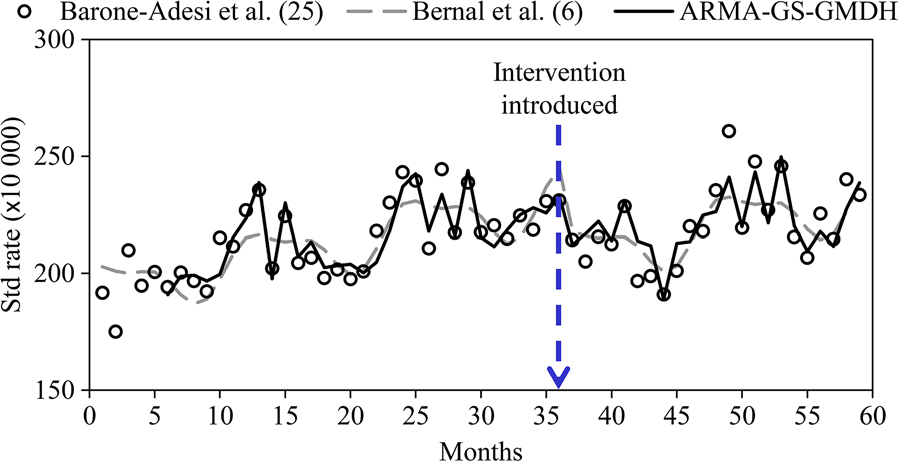
Scatter plot of observed acute coronary events (ACEs) and Auto-regressive integrated moving average–generalized structure group method of data handling (ARMA–GS-GMDH) superior hybrid model results compared to Bernal et al.’s model results. Std rate, standardized rate of ACEs [6,25]

The Taylor diagram [42] investigates the performance of the models using the standard deviation (SD) and *R* of all the tested models simultaneously. The distance from any point to the observed data in the diagram is equivalent to the centered RMSE and a precise model is one with a coefficient of determination of 1 and SD similar to the observed data. [43, 44] As illustrated in Fig. 16, the sample ITS models, including combined data-driven modeling (ARMA–GS-GMDH and ARIMA–GS-GMDH); showed a superior performance to models in the Bernal et al. study [6]. Both data-driven models were situated closer to the reference (observed) point than the models alone (GS-GMDH, ARMA and ARIMA). ARMA–GS-GMDH has a lower SD and higher *R*. By applying a combined model, the difference between the model and observed data is decreased and accuracy of predicted results is increased (Table 4, Fig. 13) in both training and testing stages. The *R* of the proposed combined data-driven model (ARMA–GS-GMDH and ARIMA–GS-GMDH) exhibited an average of 17.74% enhancement from the single stochastic model (ARMA and ARIMA) and 2.05% from the nonlinear model (GS-GMDH). Although the results of combined approaches are slightly better than the single GS-GMDH model, the accuracy is improved and the errors were about half those of the linear model.

As illustrated in Fig. 16, compared to the regression model of the Bernal et al. study [6], the single stochastic ARMA model and ARIMA have almost the same location in the diagram, in addition to showing a relatively higher *RMSE* than the single GS-GMDH and the combined model. The plot in Fig. 16 showed the superiority of the combined ARMA–GS-GMDH model with the observed ACE data [25] and the regression model [6]. Moreover, by combining the features of both models (ARMA and GS-GMDH), the fluctuations in the ACE TS could be better predicted. The series has severe fluctuations, which is why linear models alone cannot adequately forecast the data (Fig. 16). Hence, data (Table 5) showed that the combined model improved the results of the linear regression. The statistical indices indicate that the linear regression model has lower accuracy (*R* being 11.83% lower) and higher errors (*SI*, 1.33%; *MAPE*, 1.25%; and *RMSRE*, 1.19%) than the proposed model. Index *ρ* can be employed for measuring model error in addition to examining the correlation between the model and observational values. This index is lower for the ARMA–GS-GMDH model (*ρ* = 1.86%) compared to the Bernal et al. [6] model (*ρ* = 2.66%). Moreover, the Nash–Sutcliffe coefficient (*E*_*N–S*_), which is an index showing a model’s weakness in forecasting extreme values, revealed an *E*_*N–S*_ = 0.58 for the regression model which is considerably lower than the combined model *E*_*N–S*_ = 0.78.

**Table 5:** Statistical indices for the proposed model, ARMA-GS-GMDH, and segmented regression method by Bernal et al. [6] for the same testing and training periods

Model	*R*%	*SI*%	*MAPE*%	*RMSRE*%	*E*_*NS*_
Bernal et al. [6]	76.33	4.85	4.02	4.69	0.58
ARMA–GS-GMDH	88.16	3.52	2.77	3.50	0.78

*ARMA–GS-GMDH* Auto-regressive moving average–generalized structure group method of data handling, *R* correlation coefficient, *SI* scatter index, *MAPE* mean absolute percentage error, *RSMRE* root mean square relative error, *E*_*N–S*_,Nash–Sutcliffe model efficiency [6]

## Discussion

This study provides a novel approach on the use of ITS modeling based on the continuous translational data driven approach. To validate the developed model, we assessed the effects of the Italian smoking ban in public areas on hospital admissions for acute coronary events. We propose a hybrid methodology using a continuous translational data-driven approach based on a combination of the stochastic and AI methods that will (i) increase the accuracy of prediction results through a continuous modeling process, and (ii) importantly will solve a challenging issue in ITS modeling regarding the time lag between pre- and post-intervention periods, which limits the application of the segmented regression method in ITS modeling.

The complex dynamic behavior of the ACE can be modeled with a TS approach, which deduces the characteristics of the data generation process by analyzing historical data. In a recent study, Bonakdari et al. [24] showed that future prevalence a complex heath care outcome can be evaluated by historical TS at a specific time. As different dependent parameters can have a serious impact on outcome, relevant information regarding the ACE was extracted based on historical data summarized as internal patterns. In this study, the ACE TS was modeled using linear-based stochastic model (ARMA, ARIMA), nonlinear-based GS-GMDH and an integration of a continuous linear (stochastic) with a nonlinear model (data-driven method). Two fundamental premises for stochastic modeling were stationarity and normal distribution. In order to achieve stationarity, the deterministic terms should be removed from the TS. For this purpose, the structure of the TS was investigated by different tests. Initially, the ACE TS structure was investigated by stationarity and normality tests. Data showed that the TS was normally distributed but was not stationary (Table 1). The deterministic term(s) responsible for non-stationarity (trend, jump and period) terms were performed and trend and jump were found in the series. Detrending the ACE TS by trend analysis was done by stationarizing the data and then by differencing the detrended data. The former surprisingly eliminated all deterministic terms and stationarized the TS very well by 44.55% (Table 1). The latter improved the stationarity and removed the linear trend completely, made some fluctuations in the TS and increased the Fisher statistic parameter. Nonetheless, the preprocessed ACE TS was completely stationary and normal. The ARMA and ARIMA models were the first applied to the series. In order to determine the order of the models, ACF plots were used and a maximum of three parameters were required. For further investigation, ten parameters were considered in modeling. The ARMA model with seven non-seasonal auto-regressive parameters and five non-seasonal moving average parameters in the testing period outperformed the ARIMA model. For the second TS modeling approach, the ACE TS data was modeled by GS-GMDH. The most important feature of these models is their ability to model nonlinearity better than linear stochastic models. The results showed that the single nonlinear model improved the accuracy of GS-GMDH. In the third and final step, a combination of linear and nonlinear models was made. As the results depicted, both ARMA–GS-GMDH and ARIMA–GS-GMDH outperformed the single models. The ARMA–GS-GMDH model enhanced the results by an average of 17.74 and 2.03% compared to the single linear and nonlinear models. As illustrated in the Taylor diagram, combined models have a higher *R* to observed data, lower RMSE and SD closer to the observed data than other models, thus better fitting the observed data.

The proposed methodology, as well as ITS modeling, can be employed for TS prediction. To verify the performance of the methodology in TS data set modeling, another health care real case was assessed. Bhaskaran et al. [45] used TS modeling in environmental epidemiology. They studied the association between ozone levels and the total number of deaths in the city of London (UK) for a time period of five years from 1 January 2002 to 31 December 2006. In brief, the authors [45] investigated three alternative techniques including time stratified model, periodic functions, and flexible spline functions to shed light on key considerations for modeling long term patterns of studied TS. Their prediction for the total number of deaths as TS outcomes yielded a coefficient of *R* = 0.71, 0.65 and 0.69 for each method, respectively. When applying the present developed methodology to their dataset, data from the hybrid model (ARMA–GS-GMDH) give more accurate results in which *R* = 0.75 for total number of deaths. These confirm not only that the proposed hybrid model is able to predict ITS outcomes (no need to identify the implemented intervention on outcomes), but it also can be employed for modeling TS with high accuracy compared to conventional approaches.

As detailed by Bonakdari et al. [46], conventional analysis of ITS in healthcare is based on regression methods that highly depend on intervention lag time which is very often difficult to determine. However, the present methodology can continuously be employed in such cases. As examples, the hybrid model could also be applied to several health conditions and include to analyze the relationship between smoking bans and the incidence of acute myocardial infarction [47]; to analyze the quality improvement strategy on the rate of being up-to-date with pneumococcal vaccination [48]; to assess the impact of health information technology initiatives on the performance of rheumatoid arthritis disease activity measures and outcomes [16], to name a few.

As all studies, there are limitations of the hybrid methodology and mostly associated with stochastic and/or nonlinear models. The most important limitation of such a hybrid method is the minimum length of outcome TS dataset needed in the training stage. In addition, selecting appropriate parameters of stochastic models in some cases requires increasing stationarization steps which could lead to differencing, seasonal standardization, and spectral analysis methods. In turn, selecting the best input combination in nonlinear models could also be a challenging task. Finally, designing AI architecture for a given ITS requires several trial and error steps to find the appropriate parameters.

## Conclusions

Our study suggested that the proposed continuous translational data-driven model not only predicts ACEs with high accuracy and improved ITS prediction compared to current regression methods, but importantly, does not require any predefined lag time between pre- and post-intervention. This methodology can therefore be used as a reliable alternative in public health intervention evaluation. Hence, the novel hybrid approach provides a step forward by facilitating the modeling of such assessments in a short time. This is important for decision makers to manage health conditions as complex adaptive systems in a timely manner.

## Data Availability

Not relevant.

## Abbreviations

ACEs: Acute coronary events
ACF: Autocorrelation function
AI: Artificial intelligence
AICC: Akaike information criterion
AR: Autoregressive
ARMA: Auto-regressive moving average
ARIMA: Auto-regressive integrated moving average
CGA: Continuous genetic algorithm
GMDH: Group method of data handling
GS-GMDH: Generalized structure group method of data handling
IM: Inputs more
ITS: Interrupted time series
MA: Moving average
TS: Time series
KPSS: Kwiatkowski–Phillips–Schmidt–Shin
JB: Jarque–Bera
MK: Mann–Kendall
MAPE: Mean absolute percentage error
MNI: Maximum number of inputs
MNN: Maximum number of neurons
MW: Mann–Whitney
NN: Number of neurons
PD: Polynomial degree
RMSRE: Root mean squared relative error
SAR: Seasonal auto regressive
SD: Standard deviation
SMA: Seasonal moving average
SMK: Seasonal Mann–Kendall
